# Uncovering the link between malfunctions in *Drosophila* neuroblast asymmetric cell division and tumorigenesis

**DOI:** 10.1186/2045-3701-2-38

**Published:** 2012-11-14

**Authors:** Corey Kelsom, Wange Lu

**Affiliations:** 1 Department of Biochemistry and Molecular Biology, Eli and Edythe Broad Center for Regenerative Medicine and Stem Cell Research, University of Southern California, 1425 San Pablo Street, Los Angeles, CA, 90033, USA

**Keywords:** Asymmetric cell division, Neuroblasts, Polarity, Determinants, Spindle orientation, Tumorigenesis

## Abstract

Asymmetric cell division is a developmental process utilized by several organisms. On the most basic level, an asymmetric division produces two daughter cells, each possessing a different identity or fate. *Drosophila melanogaster* progenitor cells, referred to as neuroblasts, undergo asymmetric division to produce a daughter neuroblast and another cell known as a ganglion mother cell (GMC). There are several features of asymmetric division in *Drosophila* that make it a very complex process, and these aspects will be discussed at length. The cell fate determinants that play a role in specifying daughter cell fate, as well as the mechanisms behind setting up cortical polarity within neuroblasts, have proved to be essential to ensuring that neurogenesis occurs properly. The role that mitotic spindle orientation plays in coordinating asymmetric division, as well as how cell cycle regulators influence asymmetric division machinery, will also be addressed. Most significantly, malfunctions during asymmetric cell division have shown to be causally linked with neoplastic growth and tumor formation. Therefore, it is imperative that the developmental repercussions as a result of asymmetric cell division gone awry be understood.

## Introduction

Asymmetric cell division is a phenomenon that has long been studied, especially in the developing nervous system of invertebrates and vertebrates. Asymmetric cell division is a mechanism whereby any given cell divides to give rise to two daughter cells, each of which possesses a different fate than the other [1]. Such “fates” can be manifested as differences in size, morphology, gene expression pattern or the number of subsequent cell divisions undergone by the two newly born daughter cells [1].

**Figure 1:**
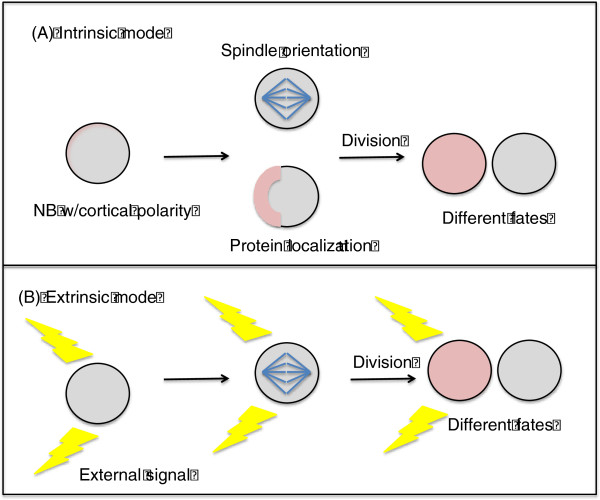
**Intrinsic vs. extrinsic modes of asymmetric cell division.** (**A**). During the intrinsic mode of asymmetric division, cells such as *Drosophila* neuroblasts possess an inherent axis of polarity. This polarity allows certain proteins such as cell fate determinants to localize asymmetrically within the cells. The mitotic spindle orients itself to be along the same axis of polarity, so when cellular division occurs, only one daughter cell receives the aforementioned determinants. Each daughter cell thus has a different fate. (**B**) During the extrinsic mode of asymmetric division, cellular precursors receive external, or extracellular, signals to self-renew (yellow). The mitotic spindle is oriented perpendicular to these external signals. When cellular division occurs, only one of the daughter cells continues to receive these signals and the two cells therefore have different fates.

To date there are two established modes of asymmetric cell division. One type of division, commonly referred to as a niche-controlled, or extrinsic, mechanism of cell division, emphasizes the importance of the stem cell niche (Figure 1A) [2]. Environmental factors influence the ability to maintain the progenitor population, and a cell relies on contact with its stem cell niche to be able to self-renew. A second, intrinsic mechanism of asymmetric cell division serves as the dominant mode of division during development and will be the focus of this discussion rather than the niche-controlled mechanism (Figure 1B). With regard to the intrinsic mechanism, regulators of self-renewal are asymmetrically localized during mitosis, so that when cells divide only one daughter cell inherits these regulators and thus takes on a different fate than its sister cell [3,4]. Actively dividing *Drosophila* neuroblasts, which serve as precursor and progenitor cells of the nervous system, take the intrinsic route of asymmetric cell division. A brief background of *Drosophila* neural progenitor cells will be given in this review. Notch signaling, which is a very important component that ties into the developmental process of neurogenesis, will also be discussed.

The major aspects of asymmetric cell division in *Drosophila* will be discussed at length. An apical-basal axis of polarity is set up within cells, which is used to both asymmetrically distribute self-renewal determinants and orient the mitotic spindle to polarize the determinants, is a very important feature of asymmetric cell division. The cell fate determinants of neural stem cell self-renewal and their asymmetric localization are also essential in ensuring that the divisional machinery operates correctly. Additionally, the role that mitotic spindle orientation plays in asymmetric division is tantamount to this developmental process and will also be discussed. The coordination of asymmetric protein localization with cell cycle progression is another aspect of asymmetric cell division that will be covered as well. Moreover, of great importance to this field of research is the concept that failure of asymmetric cell division to occur properly has widespread consequences, mainly that of neoplastic cellular growth and tumorigenesis. This review will discuss the developmental outcomes faced by dividing neuroblasts in *Drosophila* when asymmetric cell division machinery is altered or lost. In particular, the repercussions of disruption of cortical polarity due to missegregated cell fate determinants and proteins, as well as misalignment of the mitotic spindle, will be discussed. Existence of this undeniable link between asymmetric cell division gone awry and tumorigenesis shows that understanding the mechanisms behind asymmetric cell division hold great value not only on a developmental basis, but on the clinical level as well.

## Neural progenitor cells

Neuroblasts serve as the progenitor cell population in the developing Drosophila nervous system, and demonstrate the importance of asymmetric cell division in generating terminally differentiated neurons and glia. There are two types of neuroblasts in the developing nervous system – embryonic neuroblasts, which give rise to the simple nervous system present in larva, and larval neuroblasts, which generate the neurons in the fly’s adult nervous system [5,6].

**Figure 2:**
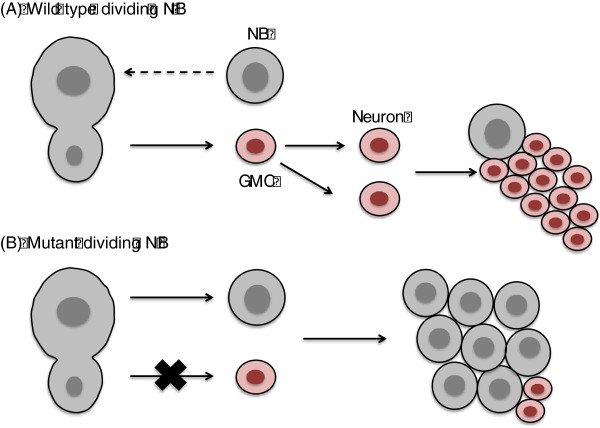
**Neuroblast self-renewal vs. differentiation and tumorigenesis.** (**A**) A wild type neuroblast divides to form two daughter cells, one of which becomes a self-renewing neuroblast (gray) and one of which becomes a ganglion mother cell (GMC) (red). The GMC divides terminally to become differentiated neurons. There is a balance between self-renewing neuroblasts and neurons. (**B**) A mutant neuroblast (such as Miranda knockouts or Pins, Lgl double knockouts) fail to divide asymmetrically and create only self-renewing neuroblasts. This results in an unrestricted growth of neural precursors at the expense of differentiated neurons, thus leading to neoplastic growth and tumor formation.

Neuroblasts initially divide symmetrically – one neural progenitor cell divides to produce two identical, daughter neuroblasts, thereby maintaining and expanding the population of neural stem cells (Figure 2A) [5,6]. As neural development progresses, neuroblasts then undergo asymmetric cell divisions. During division, one daughter cell is produced that is identical to its parent, and therefore maintains a neuroblast identity. The second daughter that is generated is smaller in size and is referred to as a ganglion mother cell (GMC). Ganglion mother cells proceed to undergo one last division to ultimately generate two differentiating neurons. As will be discussed below, actively dividing mutant neuroblasts fail to generate GMCs, resulting in the inappropriate accumulation of daughter neuroblasts at the expense of neurons (Figure 2B). In many instances this has dire effects on development.

## The role of notch signaling during neurogenesis

The Notch signaling pathway has been shown to function as a key regulator in the developing nervous system. Findings from numerous studies contribute to the fact that Notch signaling controls the balance between self-renewal and differentiation of neural progenitor cells [7-9], and elegantly coordinates neuroblastic asymmetric cell division. Neuronal differentiation is triggered by transcriptional activators such as Mash1 and Neurogenin2 (Ngn2) [10,11]. Mash1 and Ngn2 simultaneously activate the expression of the Notch receptor ligands Delta1, which activates Notch in neighboring cells. Neuralized (Neur) is an E3 ubiquitin ligase protein that facilitates the endocytosis of Delta and the extracellular domain of Notch [12-16]. Notch activation is then followed by nuclear transport of the Notch intracellular domain (NICD) and subsequent formation of a transcription activator complex [17]. This complex triggers the activation of Hes1 and Hes5, which act to repress the expression of proneural genes in neighboring progenitor cells [18]. This achieves the purpose of asymmetric cell division: it ensures the formation of differentiating neurons while simultaneously allowing neighboring cells to remain as neural progenitors.

Updates to the study of Notch signaling and its role in asymmetric cell division of neuroblasts have recently been made. Monastirioti and colleagues have identified *Drosophila* Hey as a target of Notch during neurogenesis [19]. Hey is a basic-helix-loop-helix-Orange (bHLH-O) transcription factor that is expressed primarily in the population of neuroblasts possessing activated Notch signaling, and therefore is thought to contribute to maintaining/expanding the neural progenitor cell population during development [19]. A recent study has also investigated other candidate genes that Notch signaling regulates in the context of neurogenesis. A gene referred to as Deadpan (Dpn) that encodes a bHLH transcription factor is another direct target of the Notch signaling pathway; so far this has only been demonstrated in intermediate progenitor cells [20]. Overexpression of Dpn in INPs gives rise to a cancer-like phenotype in which neuroblasts overproliferate inappropriately [20].

The first study to show the link between both asymmetric division machinery and Notch signaling and specification of a neurotransmitter neuronal phenotype was performed in *Drosophila* by Tio *et al*[21]. Findings from this study showed that loss of function of the proteins Inscuteable and Bazooka resulted in an excess number of dopaminergic neurons. On the other hand, loss of function of basally distributed proteins such as Numb resulted in a reduction of dopaminergic neurons [21]. Additionally, loss of Notch signaling results in an excess amount of this neuronal phenotype, and vice versa. Given the high levels of conservation between *Drosophila* and higher-level organisms, this study may hold great promise in understanding the link between asymmetric division and neuronal specification.

Notch signaling regulates a vast amount of developmental processes in *Drosophila*, including that of optic lobe development [22]. Briefly, loss of function analyses of both Notch and Delta demonstrate that Notch signaling might play a dual role here: maintenance of neuroepithelial stem cell population and inhibition of these stem cells toward differentiation into medulla neuroblasts [22].

## Cell-fate / segregating determinants

**Figure 3:**
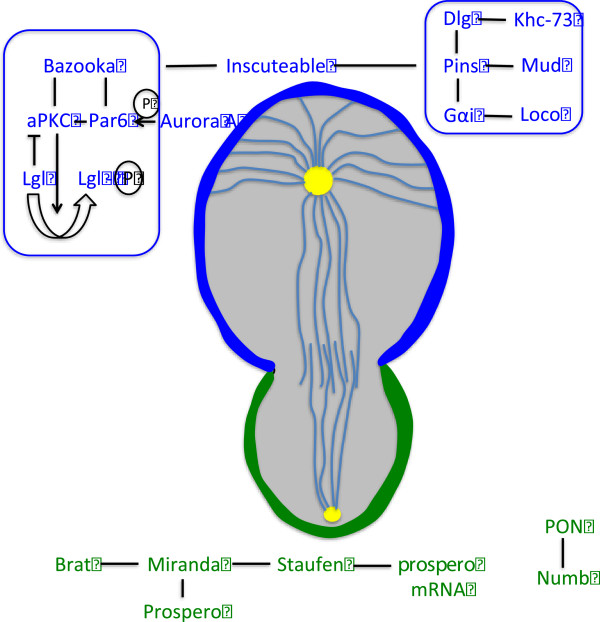
**Some of the key players in asymmetric cell division of *Drosophila *neuroblasts.** Asymmetric localization of proteins in dividing neuroblasts. Basal proteins are visualized in green, while apically localized proteins are visualized in blue. Apical proteins Bazooka, aPKC, and Par-6 form a complex whose responsibility is to establish cell polarity. There is a second complex of apical proteins, which consists of Gαi, Pins, and Loco. This complex is linked to Baz-aPKC-Par-6 by another protein known as Inscuteable. Aurora-A phosphorylates Par-6 to control aPKC’s substrate specificity. The proteins Miranda, Brat and Prospero form a complex that is basally located. PON and Numb are two other basal proteins that form a separate complex; both complexes work to regulate differentiation of the GMC.

Segregating determinants, also referred to as cell-fate determinants, are proteins that play a crucial role in specifying daughter cell fate (Table 1, Figure 3). It is the asymmetric localization of these particular determinants (in addition to other factors) to the basal side of the dividing neural progenitor cell that is largely thought to produce two daughter cells, each with a different fate.

**Table 1:** Key players in asymmetric cell division

**Asymmetric division protein**	**Protein function**	**Phenotype associated with mutation**	**References**
Numb	Neuronal differentiation	NB overproliferation	[23-27]
Pon	Neuronal differentiation	Delocalization of Numb	[28]
Brat	Regulates Prospero localization Inhibits translation	Delocalization of Prospero; NB overproliferation	[29-32]
Miranda	Localization of basal proteins	Delocalization of basal proteins; NB overproliferation	[3,33]
Prospero	Neuronal differentiation	NB overproliferation	[29-31,34-37]
Staufen	Localization of Prospero mRNA	Delocalization of Prospero mRNA	[3]
Pins, Gαi, Loco	Spindle orientation	Delocalization of basal proteins; NB overproliferation	[38-46]
	Localization of basal proteins		
Inscuteable	Links the heterotrimeric G protein complex with the Par complex		
aPKC, Bazooka/Par3, Par6	Maintaining apical and basal polarity	Apical and basal polarity defects	[28,32,33,47-57]
Mud	Spindle orientation	Spindle misorientation; NB overproliferation	[39-46,56,58,59]
Lgl (cortical localization)	Localization of basal proteins	Delocalization of basal proteins; NB overproliferation	[32,49-54]
Aurora A (centrosome)	Maintaining apical/basal polarity	Apical and basal polarity defects; spindle misorientation, NB overproliferation	[25,26,60-64]
Polo (centrosome)	Spindle orientation		
PP2A (cytoplasmic)			
Dpn	NB specification	Loss of NB	[20]
Zif	Apical/basal polarity	Apical and basal polarity defects; NB overproliferation	[55]

Cells are colored based on localization. Green denotes basal localization, blue denotes apical localization, and purple indicates nuclear localization.

### Numb

Numb is a transcription factor that was originally identified in *Drosophila* sensory organ precursor (SOP) cells and has been demonstrated to segregate asymmetrically in neuroblasts [23]. Numb has been shown to serve as a tissue-specific repressor of the Notch signaling pathway [24,65]; it binds alpha-adaptin and potentially plays a role in directing intracellular transport of Notch intermediates [66]. Loss or disruption of Numb function in the larval brain manifests in the overproliferation of mutant neuroblasts, which therefore gives rise to a tumor-like phenotype [25,26].

More recently, the role of additional proteins in regulating the asymmetric localization of Numb has been investigated. Wang and colleagues demonstrated that protein phosphatase 2A (PP2A) is a brain tumor suppressor protein that forms a heterotrimeric complex that functions to inhibit the self-renewal of neuroblasts [27]. The PP2A complex regulates, among other things, the asymmetric localization and phosphorylation of Numb [27]. Additionally, the Hem/Kette/Nap1 protein has been shown to play a very important role in the asymmetric division of *Drosophila* neuroblasts; it does so by regulating the localization of Numb and another adaptor protein known as Inscuteable [38]. Hence, *Hem/Kette/Nap1* mutant GMCs display symmetric, rather than asymmetric division. Neur, a protein previously established to play a role in Notch signaling, has recently been shown to promote the asymmetric localization of Numb by downregulating expression of the transcription factor Pdm1 [67]. This function is evidenced by mutational analaysis, which shows that Numb is symmetrically (rather than asymmetrically) localized in *Neur* mutants. Moreover, Neur overexpression results in expansion of the neuroblast population at the expense of differentiating neurons [67].

### Prospero

A second cell-fate determinant that has also been shown to segregate asymmetrically in neuroblasts is the transcription factor Prospero. Choksi and colleagues have demonstrated on the genome-wide level that Prospero (Pros) has several hundred binding sites in the *Drosophila* genome [34]. Importantly, this study showed that Pros acts as a “switch” between neuroblast self-renewal and differentiation: it has the ability to repress neuroblast and cell-cycle genes, as well as regulate neural differentiation genes [34].

*Pros* mutant GMCs fail to commit to a differentiated, neuronal fate: these mutant cells have prolonged expression of neuroblast markers and inappropriately continue to divide [34]. It had previously been postulated that the upregulation of cell cycle regulators, mainly Cyclin A, Cyclin E, and Cdc25, may be the reason for this occurrence in *Pros* mutant neuroblasts [35]. More recently, Berger *et al* have demonstrated that Cyclin E possesses cell-cycle independent roles in asymmetric division: it inhibits Pros function, and may also serve to regulate the cortical localization of Pros, hence allowing neuroblasts to maintain their identity rather than committing to the neuronal lineage [36]. By regulating Pros localization, CycE therefore plays a crucial role in maintaining the neural progenitor population. Similar to Numb, mutational analysis shows that *Pros* mutations give rise to stem cell-derived tumors in larval neuroblasts [29-31].

In addition to its role as a cell fate determinant in GMCs, Pros also possesses a role in coupling cell cycle progression to neurogenesis during development: its transient expression ensures that neuronally-committed cells exit the cell cycle at the appropriate time [37].

### Brat

Brat is the third cell-fate determinant and growth inhibitor that was discovered to play a role in regulating the balance between neuroblast self-renewal and differentiation [29-31]. During neural development, Brat (in combination with Pros) segregates asymmetrically into only one of two daughter neuroblasts to specify GMC fate [30]. Similar to Numb, loss of Brat results in both daughter cells taking on a neuroblast identity, which ultimately gives rise to a tumor phenotype [30]. Further mutational analyses have demonstrated that the phenotype of *Pros/Brat* double mutants is one that lacks most, if not all, GMCs due to an overexpansion of neuroblasts [30]. Observations of these mutant phenotypes have led to the speculation that Brat may function to inhibit cell growth in one of two newly born neuroblast daughters so as to generate one neuron and one neuroblast, rather than two neuroblasts [32]. The true molecular mechanism by which Brat operates, however, remains to be clarified.

## Adaptor proteins

While proper function of the segregating determinants is crucial for asymmetric cell division, adaptor proteins are just as important in ensuring that division is properly executed. Adaptor proteins facilitate the asymmetric localization of Numb, Pros and Brat.

### Miranda

Miranda serves as the adaptor protein for segregating determinants Pros, Brat, and Staufen, although Staufen’s functions will not be covered in this review. For more details regarding Staufen, refer to Betschinger and Knoblich [3]. Miranda behaves similarly to Pros and Brat; in dividing neuroblasts it too localizes asymmetrically and segregates into one of the two daughter cells [3]. Importantly, when Miranda is mutated, both Pros and Brat segregate symmetrically rather than asymmetrically in dividing neuroblasts, and the cell-fate determinants are therefore uniformly cytoplasmic.

Atwood and colleagues have more recently shown that atypical protein kinase (aPKC), which is a regulator of cell polarity, directly phosphorylates Miranda [33]. Phosphorylation displaces Miranda from the apical cortex, where it can then work to polarize the cell-date determinants. These findings counter the theory that Miranda is regulated by a more complicated cascade involving aPKC, Lgl and myosin II [33]. Regardless of the mechanism by which it works, Miranda is a crucial adaptor protein that connects Pros and Brat to the machinery for asymmetric protein localization.

### Pon

Pon, which stands for Partner of Numb, is so-named because of its function: it is the adaptor protein for Numb and therefore binds to Numb. Unlike Miranda, however, Pon is not required for the asymmetric localization of Numb [28]. In the absence of Pon, localization of Numb is delayed in metaphase, which therefore results in a defect in the neuroblast self-renewal [28].

## Setting up polarity

While the asymmetric localization of cell-fate determinants and the functions they play in neuroblast self-renewal or neuronal commitment has been established, the question of how they are directed to the basal cortex remains to be answered. The answer lies within an axis of polarity that is set up during interphase. Cell-fate determinants and the orientation of stem cell division both take instruction from this axis of polarity, which consists of aPKC and the Par proteins Par-3 (Bazooka in *Drosophila*) and Par-6 (Figure 3) [47,48].

Par-3, Par-6 and aPKC are required for establishing apical-basal polarity in developing neuroblasts: they concentrate to the apical cell cortex of the neuroblast [32]. The localization of these proteins is *opposite* of the location that the cell-fate determinants concentrate in mitosis, and their presence ensures that the determinants are segregated into the basal cell cortex. When any one of the three polarity proteins is mutated, the cell-fate determinants are distributed uniformly in the cell cortex, and mitotic spindles orient randomly [47,48]. Hence, aPKC and the Par proteins are critical for constructing a “blue-print”, in which the cell-fate determinants are properly distributed and the mitotic spindle is properly oriented.

Due to the extensive amount of literature in existence concerning the role of the Par proteins, this discussion will mainly emphasize the role that Lgl, the substrate of aPKC, plays in the asymmetric localization of segregating determinants.

Lethal (2) giant larvae (abbreviated Lgl) was identified by several groups as the key substrate for aPKC [49-51]. Unlike the other polarity proteins that are apically localized, Lgl is uniformly distributed throughout the cortex. Lgl is necessary for ensuring that the cell-fate determinants are brought to the cell cortex and localized asymmetrically during mitosis [52,53]. The mechanism by which Lgl is able to achieve its purpose was elucidated soon after, and concerns the Par proteins, mainly aPKC. aPKC is responsible for phosphorylating Lgl on three conserved serines at the cell cortex [49]. This phosphorylation event is proposed to prevent Lgl from associating with the actin skeleton, and most significantly, prevents the cell fate determinants from localizing apically [49]. aPKC-mediated phosphorylation of Lgl seems to somehow inactivate Lgl, and is evidenced by the phenotype of aPKC overexpression, which resembles that of Lgl loss of function.

Adding more complexity to the puzzle is the fact that aPKC also possesses the ability to phosphorylate segregating determinants directly, rather than acting on Lgl first. Smith and colleagues have demonstrated that aPKC can directly phosphorylate Numb, whereby Numb is transported from the cell cortex into the cytoplasm [54]. This study demonstrated, utilizing a form of Numb lacking two protein kinase C (PKC) phosphorylation sites, that inability to phosphorylate Numb results in the inappropriate accumulation of Numb at the cell membrane and unresponsiveness to PKC activation [54].

In a 2008 review, Knoblich has proposed a model to account for aPKC and Lgl activity in neuroblasts, whereby aPKC, which is localized at the apical cortex, functions to restrict Lgl to the basal side of the neuroblast [32]. Given Lgl’s responsibility of recruiting the cell-fate determinants to the cortex, this makes sense, since the determinants only localize basally. While this model is certainly logical, the molecular mechanism by which Lgl operates has not yet been elucidated.

Recent studies have delved into mechanisms underlying aPKC function. Chang *et al*, for instance, have shown that Zif is a transcription factor and that Zif is required for aPKC to both be expressed and asymmetrically localized [55]. Zif acts to directly repress transcription of aPKC, and in turn, aPKC phosphorylates Zif, which ultimately leads to Zif inactivation in neuroblasts. The combined actions of these two proteins thus play an indispensable role in setting up cortical polarity and also controlling progenitor self-renewal [55].

## Importance of proper mitotic spindle orientation

The significance of mitotic spindle orientation in regulating neuroblast division has also been extensively studied and renewed [68,69]. Here, the coordination between mitotic spindle orientation and asymmetric localization of the segregating determinants will be discussed.

Kraut and colleagues first determined that a protein known as Inscuteable (Insc) plays an immensely important role in coordinating mitotic spindle alignment and localization of the cell-fate determinants to the basal cortex in dividing neuroblasts [70]. Inscuteable operates by binding the polarity protein Bazooka (Par-3) in the apical region of neuroblasts, and recruits another protein called Pins (which will be discussed below) [39]. Zhu and Bhat have recently shown that the Drosophila protein Hem/Kette/Nap1 also regulates localization of Inscuteable. As was previously discussed, this protein regulates the asymmetric division of neural progenitors by controlling Numb localization [38].

It is important to note that the binding of Insc to Pins triggers the activation of two downstream pathways that participate in mitotic spindle positioning, both of which are mediated by Pins. The first pathway is often referred to as the Pins-Mud pathway. Pins is a protein whose structural features are functionally purposeful: it contains three domains called GoLoco domains in its C-terminal region; these domains bind Gαi, which is a heterotrimeric G protein subunit [39]. Upon binding, Pins is recruited to the plasma membrane and switches from and switches from an inactive to active state. It is in this active state that the N-terminal region of Pins binds to another protein called Mud (which stands for Mushroom body defect) [40-42]. Mud is the *Drosophila* homolog of NuMA, and is thought to play a role in recruiting the Dynein/Dynactin complex [43]. This complex functions to generate pulling forces on astral microtubules so as to further advance mitotic spindle positioning [43].

The second pathway that is activated upon Insc binding Pins is the Dlg pathway [44,45]. In a 2005 study, Siegrist and Doe elegantly showed that astral microtubules bind to a kinesin referred to as Khc-73 as well as the protein Discs large (Dlg). Of note is that the Dlg pathway functions during metaphase to coordinate neuroblast polarity with the mitotic spindle, independent from the Pins-Mud pathway [45].

Recently, others have further investigated the mechanisms by which Inscuteable exerts its effects on mitotic spindle positioning through Pins. Results from a study by Mauser and Prehoda have suggested that Insc preferentially inhibits the Mud pathway, while enabling continued activation of the Dlg pathway [46]. A variety of rationales may explain these findings, one of them being assurance that the spindle is attached to the cortex via Dlg before spindle pulling forces are activated via the Mud pathway [46].

A 2011 study has characterized the *Drosophila* cytoplasmic polyadenylation element binding (CPEB) protein Orb2. CPEB proteins function to bind mRNAs in order to control their localization and subsequent translation. Hafer and colleagues report that Orb2 functions in the asymmetric division of both stem and precursor cells in the context of the developing *Drosophila* nervous system [56]. Additionally, *Orb2* mutants present with disrupted mitotic spindle alignment; results from this study suggest that it may serve to promote the localization of aPKC [56].

Speicher *et al* were first to demonstrate that the PDZ protein Canoe (Cno) plays a role in both the localization of cell-fate determinants and orientation of the mitotic spindle in asymmetrically dividing neuroblasts [58]. Cno apically localizes with the Bazooka/Par-3 in neuroblasts, and was also found to be essential for proper distribution of cell-fate determinants on the basal side of the cell; importantly, failure of the determinants to basally distribute resulted in misorientation of the mitotic spindle. This study further demonstrated that Cno interacts with the proteins Inscuteable, Gαi, and Mud, and acts downstream of apical proteins Insc-Pins-Gαi, but upstream of Mud [58]. More recently confirming Cno’s involvement in regulating mitotic spindle orientation and neuroblast cortical polarity is a study demonstrating that Rap1, a Ras-like small guanosine triphosphatase, signals through Cno and another guanine nucleotide exchange factor known as Rgl in order to regulate neuroblast polarity [59]. Carmena and colleagues postulate that Rap1 forms a novel Rap1-Rgl-Ral signaling network that interacts with other apical proteins to influence neuroblast cortical polarity and spindle orientation: loss of function of Rap1, Rgl and Ral proteins affect both spindle orientation and the localization of proteins Mud and Cno [59].

## Consequences of disrupting asymmetric cell division – neoplastic growth and tumorigenesis

It has long been postulated that tumorigenesis and uncontrolled cellular replication may be causally linked to asymmetric cell division gone awry. To date, transplantation studies in *Drosophila* (as well as in mammals) remain the most reliable method for assessing the neoplastic cellular growth of cells in the context of experimentally induced tumors [71]. When tissue possessing mutant forms of the proteins central to asymmetric neuroblast division, such as Miranda, Prospero, Numb, Lgl, Brat and Pins, is transplanted into the wild-type *Drosophila* hosts, excessive overgrowth is observed; this massive cellular overgrowth is lethal to the organism [72,73]. Further analysis shows that the phenotypes observed from these transplantation experiments resemble malignant, neoplastic growth. The tumors that grow in these organisms have the capacity to be reimplanted into successive healthy host organisms for a number of years, demonstrating that these cells are immortalized [73]. Importantly, these transplanted cells seem to exhibit metastatic behavior: these cells are capable of migrating away from the site of initial tumor growth, can migrate through a number of cell layers, and can form secondary colonies [71,73]. Transplantation of tissue possessing mutations for asymmetric division is also a contributing factor for genome instability: alterations in centrosome morphology and number are observed in addition to cytologically abnormal karyotypes [72,73]*.* These observations clearly demonstrate a commonality – the disruption of asymmetric division in neuroblasts, resulting in the overgrowth of self-renewing daughter cells at the expense of neuronal daughters.

The missegregation of apically- and/or basally localized proteins is a major causal factor for neoplastic growth and tumorigenesis in neuroblasts. Take, for instance, brain tissue possessing neuroblasts with mutated versions of Pins, Miranda, Numb, or Prospero, all crucial asymmetric cell division proteins: transplantation experiments with such brain tissue invariably results in inappropriate neuroblast overproliferation and gives rise to a cancer-like phenotype and ultimately, death [74]. Interestingly, one particular study generated a *Drosophila* transplantation model of neural stem cell-derived cancer [75]. RNAi-mediated knockdown of cell fate determinants Numb, Brat, and Prospero in neuroblasts resulted in neoplastic tumor formation after transplantation [75]. Additionally, a plethora of studies have demonstrated that absence or disruption of proper cell-fate determinant function (Numb, Pros, Brat) results in an uncontrollable expansion of the neuroblast/progenitor pool, and heavy (or complete) depletion of neuronally committed cells [25,26,28-31,34].

Mutations in three genes in particular, Dlg, Lgl, and Scribble (Scrib), also results in the inability of cell fate determinants to localize asymmetrically in neuroblasts [30,52,53,57]. These mutations were also responsible for inducing the formation of neoplastic tumors within the nervous system [30,52,53,57]. As was previously mentioned, Lgl in particular serves to restrict aPKC to the self-renewing apical daughter cell [57]. Simultaneous disruption of both Pins and Lgl proteins results in unrestricted growth of neuroblasts due to the delocalization of aPKC [57]. Results from this same study demonstrated that overexpression of a membrane-targeted form of aPKC results in a significant increase in number of neuroblasts [57]. The opposite is also true: loss or reduction of aPKC expression results in a corresponding reduction of neuroblast numbers [57,76].

Because aPKC clearly serves an important role in maintaining a balance between neuroblast self-renewal and differentiation, it is logical to determine which molecules or factors regulate aPKC itself. Two previously mentioned proteins, Protein phosphatase PP2A and the zinc finger protein known as Zif, have been shown to negatively regulate aPKC [27,55]. Zif binds to a region of aPKC, thereby repressing aPKC transcription [55]. There is, however, a more complicated feedback mechanism between aPKC and Zif that has yet to be completely elucidated in the context of regulation of neuroblast self-renewal. As these studies have shown, it is therefore of utmost importance that these determinants (and their adaptor proteins) segregate properly so as to maintain the pool of GMCs that eventually give rise to the differentiating neurons of the developing nervous system.

Just as the loss of segregating determinants leads to overgrowth and tumor formation, so too will the loss of machinery controlling mitotic spindle orientation. Several studies have demonstrated that improper mitotic spindle orientation causes improper segregation of proteins that are normally asymmetrically localized. The improper segregation of said proteins in turn leads to unrestricted neuroblast division and outgrowth. As was previously mentioned, loss of Mud, a protein crucial for orienting the mitotic spindle, results in abnormal proliferation of neuroblasts [40-42]. Additionally, neuroblasts possessing double mutations for *Lgl* and *Pins*, as well as *Dlg/Gβγ* double mutants (Gβγ is a cortically localized protein that also regulates spindle orientation), demonstrate significant overproliferation relative to wild-type [57,77].

Additionally, in a 2009 study, Cabernard and Doe attempted spindle disruption without altering cell polarity via live imaging of polarity markers and spindle orientation over a time period of multiple divisions, then analyzed cell fate utilizing molecular markers [78]. Results from this study suggest that when the spindle is oriented orthogonally to apical-basal polarity, the cell-fate determinants fail to localize symmetrically rather than asymmetrically, and both daughter cells ultimately form neuroblasts [78]. Maintaining mitotic spindle orientation is thus crucial for maintaining the neuroblast population, ensuring that differentiating neurons are formed and preventing unwanted tumor formation.

Most recently, a protein known as Huntingtin (abbreviated Htt) has been shown to regulate mitotic spindle orientation in *Drosophila* neuroblasts as well as in mammalian cortical progenitor cells [79]. Htt is mutated in Huntington’s disease, a neurodegenerative disorder caused by a genetic defect on chromosome 4. Godin *et al* showed that RNAi-mediated knockdown of Htt prevents proper spindle orientation; this occurs due to the incorrect localization of the p150^*Glued*^ subunit of dynactin, dynein, and the NuMA protein [79]. In addition to demonstrating its role in controlling mitosis, further elucidation of Htt’s role may hold great promise in the therapeutic treatment of Huntington’s disease.

Evidence is emerging that cell cycle regulators play an important role in the division of *Drosophila* neuroblasts, and disrupting their function may give rise to tumorigenesis. Cell cycle genes have been shown to act as tumor suppressors, whose loss of function manifests in the inability to carry out asymmetric protein localization [80]. Mutations in cell cycle-related genes has also been shown to affect the specification of daughter cell fate, as well as the inappropriate self-renewal of neural progenitors rather than differentiation [80]. A crucial study that first began to characterize the involvement of cell cycle regulators in asymmetric cell division focused on the role of a dominant-negative allele of Cdc2, known as *cdc2,cdc2*^*E51Q* 76^. In this study, a genetic screen was utilized to identify mutations that inappropriately transformed asymmetric GMC divisions into symmetric divisions, which produced two identical neuronal daughter cells at the expense of GMC daughters. Cdc’s normal function is to form a complex with cyclins in order to activate CDK1, a kinase required to force cells from G2 phase to mitosis. In the case of the *cdc2,cdc2*^*E51Q*^ mutant isolated from the genetic screen, both apical and basal proteins involved in neuroblast asymmetric division were unable to localize symmetrically, resulting in the conversion of asymmetric division to symmetric division [81]. Furthermore, utilization of a temperature-sensitive *Cdc* mutant, in which proper Cdc function was weakened, generated the same results [81]. This study clearly demonstrated that cell cycle genes and the proteins they encode should be taken into account when analyzing the underlying mechanisms of excessive cellular growth.

Two other cell cycle regulators are Aurora-A (abbreviated Aur-A) and Polo, which are serine/threonine kinases. Aur-A has been shown to inhibit the self-renewal of *Drosophila* larval neuroblasts and promote neuronal differentiation [25]. Mutational analyses demonstrate that *Aur-A* mutant neuroblasts undergo unrestricted self-renewal, and this phenotype is due to both abnormal aPKC/Numb cortical polarity and misalignment of the mitotic spindle [25]. Another study corroborates these findings: an excessive number of neuroblasts is observed in *Aur-A* mutants, thereby showing that Aur-A acts as a tumor suppressor [26]. A later study revealed the molecular mechanism for the asymmetric localization of Numb: Aur-A phosphorylates Par-6, which in turn activates aPKC and phosphorylates Lgl; this even allows Bazooka to enter the complex, thereby allowing aPKC to regulate Numb localization to one side of the cell cortex [60]. Polo is another cell cycle regulator that has a hand in asymmetric cell division. Like Aur-A, Polo also acts as a tumor suppressor: excessive neuroblast numbers are observed in *Polo* mutants [28]. Wang and colleagues have demonstrated that Polo phosphorylates Partner of Numb (Pon), and this is important for Pon to be able to localize Numb [28].

What must also be taken into account are the phosphatases that counteract the effects of the aforementioned kinases. Protein phosphatase 4A (PP4A) and protein phosphatase 2A (PP2A) also play a role in the division of *Drosophila* neuroblasts. The mechanisms by which these phosphatases operate to regulate are more complicated [61-63] and will not be discussed here; for more details refer to the review by Chang et al [64].

As was mentioned earlier, the missegregation of apically- and/or basally-localized proteins is tantamount in ensuring that the asymmetric division machinery function without fail. Slack and colleagues have elegantly demonstrated that anaphase-promoting complex/cyclosome (APC/C) function is necessary for the proper asymmetric localization of Miranda in particular, as well as its cargo proteins [82]. The APC/C is a protein complex whose function is required in the context of the cell cycle; it has been shown to possess roles in destruction of mitotic cyclins, regulation of DNA replication, centrosome, duplication, and mitotic spindle assembly [83-86]. Other recent studies have demonstrated that APC/C also has a hand in non-cell cycle-related functions [87-89]. Most relevant to this discussion, Slack *et al* demonstrated that when any of the core components of APC/C are mutated, Miranda and its cargo proteins, Prospero, Brat and Staufen, are unable to properly localize, and instead localize in a pericentrosomal region [82]. Miranda possesses a putative APC/C motif at its C-terminal region; disruption or absence of this region results in an inability to ubiquitinate Miranda [82]. Further analysis showed that APC/C may facilitate ubiquitination of Miranda, and that this ubiquitination event may be required for the proper asymmetric localization of this protein in dividing neuroblasts [82]. Although much progress has been made in terms of understanding how cell cycle is coupled to asymmetric division machinery, a more widespread and thorough analysis should be performed to identify candidate cell cycle genes involved asymmetric cell division.

## Concluding remarks

Although asymmetric cell division plays a role in the developmental processes of many organisms, it has been best studied in the context of neurogenesis in *Drosophila* neuroblasts. While the molecules and proteins that play roles in setting up cortical polarity and spindle orientation have been heavily studied and documented, the utilization of neuroblasts as a model to study uncontrolled stem cell self-renewal, and ultimately, tumorigenesis, is relatively new. It is clear that impinging upon any of these components of the asymmetric division machinery will have dire consequences. Transplantation models such as that utilized by Laurenson *et al* are likely to provide insights into the connection between asymmetric division and tumorigenesis.

A “big picture” approach to understanding the carefully controlled balance between neural stem cell self-renewal and differentiation has utilized transgenic RNAi on the genome-wide level to identify over 600 genes that may control this self-renewal vs. differentiation switch [90]. Knockdown of key genes, such as transcription factors Lola, Ssrp and Barc, results in defective neuroblast lineages. The identification of genes in this study should be further studied with the objective of creating functional gene networks that interact or influence asymmetric cell division machinery.

Despite the advances that have been made in this field of research, there are many questions that have yet to be answered. For one, the exact time point at which neuroblasts re-enter the cell cycle at the end of the embryonic stage remains unclear. Additionally, one must consider the fact that asymmetric cell division in mammalian systems opens a whole new door of complexity: although there may be a high degree of conservation between *Drosophila* and mammalian homologs, the roles that these mammalian homologs play may be not at all similar to that of the cell fate determinants and proteins in *Drosophila*. Gaining an understanding of the similarities and differences between *Drosophila* neuroblasts and mammalian neural progenitors may hold the key to understanding and/or treating various neurological degenerative disorders.

## Competing interests

The authors declare that they have no competing interests.

## Authors' contributions

Both CSKK and WL drafted the manuscript. Both authors read and approved the final manuscript.
